# Lipemic serum in hyperlipidemic pancreatitis

**DOI:** 10.1186/1757-1626-2-198

**Published:** 2009-11-18

**Authors:** Konstantinos Michalakis, Eleni Basiakou, Theodoros Xanthos, Panagiotis Ziakas

**Affiliations:** 1 NIH, Endocrine Department, Bethesda, Maryland, USA; 2 University of Athens Medical School Department of Pathophysiology and Experimental Surgery and Surgical Research, Greece

## Abstract

**Background:**

A 37 year-old man presented to the emergency department complaining of six hour diffuse abdominal pain accompanied by persistent vomiting.

**Case report:**

The patient had a heavy meal a few hours before. There were no signs of peritonitis. Routine laboratory examinations revealed leukocytosis, hyperglycemia and hyperamylasemia (serum amylase: 380 mg/dl, urinary amylase: 1150 mg/dl).

**Conclusion:**

The lipid profile revealed an impressive elevation of triglycerides (4800 mg/dl) and cholesterol (1009 mg/dl) levels. The serum was extremely lipemic. The abdomen computed tomography confirmed the diagnosis of pancreatitis.

## Document

**Figure 1:**
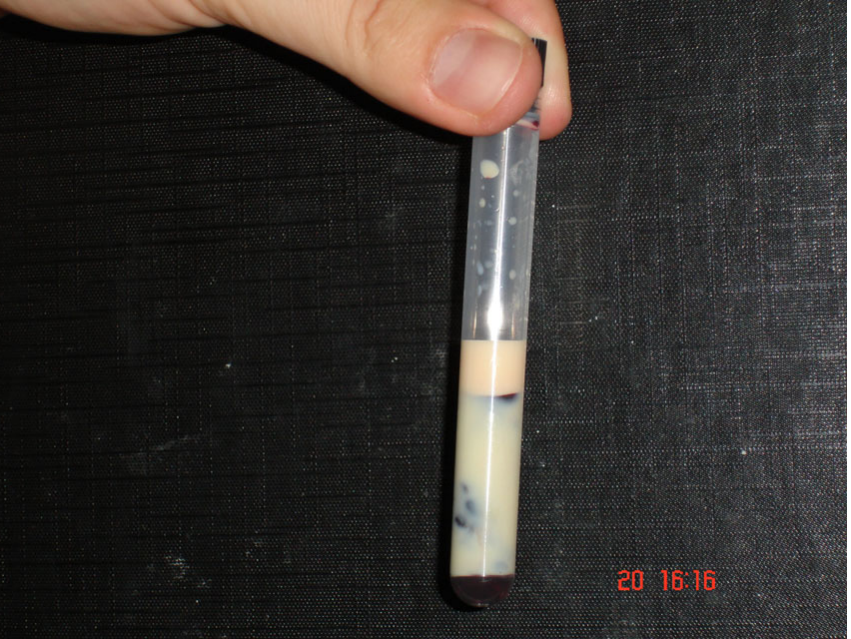
**Lipemic serum- picture captured 10 minutes after blood was drawn**.

A 37 year-old man presented to the emergency department complaining of six hour diffuse abdominal pain accompanied by persistent vomiting. The patient had a heavy meal a few hours before. His past personal history was positive for heavy alcohol consumption, but there was no known history of diabetes mellitus. The patient reported that his father suffered from hypercholesterolemia controlled by a low dose of statin. The patient's physical examination revealed normal bowel sounds and a mild diffuse abdominal tenderness. There were no signs of peritonitis. The rest of the clinical examination was without abnormal findings. His vital signs were: Blood Pressure: 160/90 mmHg, Heart Rate: 86 bpm. Respiratory Rate: 26 breaths/min, Rectal Temperature: 37,1°C Pulse Oximetry: 95% (on air). Routine laboratory examinations revealed leukocytosis (WBC: 13,3 K/μl), hyperglycemia (378 mg/dl) and hyperamylasemia (serum amylase: 380 mg/dl, urinary amylase: 1150 mg/dl). BMI was 29 kgr/m^2^. The lipid profile revealed an impressive elevation of triglycerides (4800 mg/dl) and cholesterol (1009 mg/dl) levels. The serum was extremely lipemic (Figure 1). The abdomen computed tomography confirmed the diagnosis of pancreatitis. The patient was admitted for further evaluation and treatment. He was managed conservatively with lipid-lowering medications (atorvastatin, fenofibrate), hydration and dietary restriction. The patient was treated with rapid acting insulin for 2 days during admission. Moreover, he underwent two sessions of plasmapheresis, in order to achieve quick and acute improvement, but was never admitted to the ICU. His clinical condition improved significantly and the patient was discharged in good health five days later, with a triglyceride level of 420 mg/dl. Alcohol consumption was the secondary releasing factor of this episode of acute pancreatitis.

Hypertriglyceridemia is a relatively uncommon cause of pancreatitis [1]. Pancreatitis secondary to hypertriglyceridemia typically presents as an episode of acute pancreatitis (AP) or recurrent pancreatitis and rarely as chronic pancreatitis [1,2]. An important and usually identifiable risk factor is a serum triglycerides level of 1000-2000 mg/dl in a patient with an already known type I, IV or V hyperlipidemia (Fredrickson's classification). The typical clinical profile is a patient presenting with a preexisting lipid abnormality in combination with a secondary co-releasing factor, such as poorly controlled diabetes mellitus, medications related to induction of AP or alcohol consumption. In addition to the usual findings in AP, in Hyperlipidemic Pancreatitis (HLP), the serum pancreatic enzymes may be normal or slightly elevated [3], even in the presence of severe AP that has been confirmed by imaging techniques [2,4]. The diagnostic hallmark consists of a mildly elevated amylase with interestingly low urine amylase levels along with the presence of lipemic serum in a patient with a family history of disturbed lipid profile [1]. As far as it concerns the clinical course of HLP, it does resemble to that of AP derived from other causes, with similar complications but not in a higher rate than in other types of AP. Routine management should not differ much, aiming at a reduction of triglyceride levels to less than 1000 mg/dl, through dietary restriction of fat and lipid-lowering medications (mainly fibric acid derivatives). Such a reduction has been proved to prevent from recurrent episodes of AP, whereas there are anecdotal reports of other therapies, such as plasmapheresis.

## Consent

Written informed consent was obtained from the patient for publication of this case report and accompanying images. A copy of the written consent is available for review by the Editor-in-Chief of this journal.

## Competing interests

The authors declare that they have no competing interests.

## Authors' contributions

KM is the physician that was responsible for the patient during his stay at the University Hospital. PZ is the haematologist that consulted for serum parameters. EB and TX are responsible for the laboratory evaluation of the patient. All authors read and approved the final manuscript.

## References

[B1] Linares LC Pelletier AL Czernichow S Vergnaud AC Bonnefont-Rousselot D Levy P. Acute pancreatitis in a cohort of 129 patients referred for severe hypertriglyceridemia Pancreas 2008 37 1 13 2 10.1097/MPA.0b013e31816074a1 18580438

[B2] Tsuang W Navaneethan U Ruiz L Palascak JB Gelrud A Hypertriglyceridemic pancreatitis: presentation and management Am J Gastoenterol 2009 104 4 984 91 10.1038/ajg.2009.27 19293788

[B3] Frank B Gottlieb K "Amylase normal, lipase elevated: Is it pancreatitis? A case series and review of the literature" Am J Gastroenterol 1999 94 2 463 469 10022647 10.1111/j.1572-0241.1999.878_g.x

[B4] Yadav D Pitchumoni CS "Issues in Hyperlipidemic pancreatitis" J Clin Gastroenterol 2003 261 1 54 62 10.1097/00004836-200301000-00016 12488710

